# Assessing Mortality Prediction: Comparing the Effectiveness of the Fournier Gangrene Severity Index and the Uludag Index

**DOI:** 10.7759/cureus.104290

**Published:** 2026-02-26

**Authors:** Emma Berenice López Pacheco, Hugo Fernando Narvaez Gonzalez, Martha Asunción Sánchez-Rodríguez, Omar López Osorio, Arcenio Luis Vargas Avila

**Affiliations:** 1 General Surgery, Hospital Regional General Ignacio Zaragoza, Instituto de Seguridad y Servicios Sociales de los Trabajadores del Estado (ISSSTE), Mexico City, MEX; 2 Gastrointestinal Endoscopy, Centro Médico Nacional 20 de Noviembre, Instituto de Seguridad y Servicios Sociales de los Trabajadores del Estado (ISSSTE), Mexico City, MEX; 3 Surgery, Hospital de Especialidades de la Cuidad de México Dr. Belisario Domínguez, Mexico City, MEX; 4 Gerontology Research Unit, Facultad de Estudios Superiores Zaragoza (FES) Universidad Nacional Autónoma de México (UNAM), Mexico City, MEX; 5 Surgery, Hospital Regional General Ignacio Zaragoza, Instituto de Seguridad y Servicios Sociales de los Trabajadores del Estado (ISSSTE), Mexico City, MEX

**Keywords:** fournier gangrene severity index, fournier’s gangrene (fg), mortality index, risk stratification, uludag fournier gangrene severity index

## Abstract

Background

Fournier’s gangrene (FG) is a fulminant necrotizing infection associated with substantial morbidity and high mortality. Early and accurate risk stratification is essential to guide clinical decision-making. The Fournier Gangrene Severity Index (FGSI) and the Uludag Fournier Gangrene Severity Index (UFGSI) are widely used prognostic tools; however, their comparative performance in Mexican populations remains insufficiently characterized. This study aimed to assess the association of FGSI and UFGSI with in-hospital mortality and to explore their comparative prognostic performance in a Mexican cohort.

Methodology

This retrospective cohort study was conducted at a tertiary-level public hospital in Mexico City. We included 65 adults with confirmed FG treated between July 2022 and February 2025. Clinical, demographic, and laboratory data were collected to calculate FGSI, UFGSI, and the Age-Adjusted Charlson Comorbidity Index. Survivors and non-survivors were compared using Student’s t-test and chi-square tests, and associations with in-hospital mortality were evaluated using multivariate logistic regression with traditional and adjusted cutoff values. Statistical significance was defined as a p-value <0.05.

Results

Overall mortality was 18/65 (27.7%). Most patients were male (44/65, 67.7%), and the mean age was 59.4 ± 11 years. Non-survivors were older than survivors (64.8 ± 10 vs. 57.5 ± 11 years; p < 0.05). The most frequent etiology was proctologic origin (37/65, 56.9%). The most common comorbidity was diabetes (46/65, 70.8%), followed by systemic arterial hypertension (25/65, 38.5%). Mean index scores were higher among non-survivors than survivors for both FGSI (9.1 ± 4 vs. 5.8 ± 3; p = 0.002) and UFGSI (12.6 ± 5 vs. 8.4 ± 4; p = 0.001). Using traditional cutoff points, multivariate logistic regression demonstrated statistically significant associations with mortality for FGSI (odds ratio (OR) = 3.70; 95% confidence interval (CI) = 1.01-13.55; p = 0.048) and UFGSI (OR = 4.70; 95% CI = 1.26-17.44; p = 0.021).

Conclusions

FGSI and UFGSI were both associated with in-hospital mortality in FG. In this cohort, UFGSI showed a stronger association with in-hospital mortality using traditional cutoff values. Larger prospective studies are warranted to validate adjusted cutoff points across diverse clinical settings.

## Introduction

Fournier’s gangrene (FG) is a severe, rapidly progressing infectious disease characterized by high morbidity and an alarmingly elevated mortality rate, which can reach up to 67%. It predominantly affects males, with a male-to-female ratio of approximately 10:1, and commonly occurs in individuals aged 50 to 70 years. The infection primarily targets the perineal region and external genitalia, spreading swiftly along fascial planes, leading to arterial thrombosis and tissue necrosis. These pathological processes may culminate in multisystem organ failure, shock, and, ultimately, death [1-3].

The sources of infection are diverse, including colorectal, dermatological, genitourinary, gastrointestinal, or mixed origins. Comorbid conditions such as diabetes, chronic alcoholism, immunosuppression, obesity, and poor hygiene have been linked to the development of FG, as they create an environment conducive to bacterial proliferation [1-6].

Several factors influence patient mortality in FG, notably age, delayed diagnosis, and the presence of comorbidities such as renal or cardiovascular disease. To evaluate the impact of these comorbidities on clinical outcomes, the Age-Adjusted Charlson Comorbidity Index (ACCI) is commonly used. This tool considers 19 chronic medical conditions, assigning weighted scores, and includes additional points based on patient age. Although not initially designed for FG, the ACCI has demonstrated utility as a prognostic adjunct [3,6-11].

To enhance prediction of disease severity and improve risk-stratification accuracy, tailored scales such as the Fournier’s Gangrene Severity Index (FGSI) and the Uludag Fournier’s Gangrene Severity Index (UFGSI) have been developed [1].

The FGSI was established in 1995 to evaluate clinical and laboratory parameters, such as temperature, heart rate, respiratory rate, serum sodium, potassium, creatinine, leukocyte count, hematocrit, and bicarbonate, and to assign scores that reflect the patient’s severity. A recent meta-analysis by Tufano et al. (2023) included 40 studies with 2,257 patients and reported sensitivities of 84% and specificities of 85%. A score exceeding 9 is associated with a 75% probability of death, whereas a score of ≤9 is associated with a 78% survival probability [8-12].

In 2010, Yilmazlar and colleagues developed the UFGSI, which incorporates the extent of tissue involvement and age as additional variables. This scale demonstrated a sensitivity of 91.7% and a specificity of 70.4%. A score of ≤9 predicts an 81% chance of survival, while scores greater than 9 indicate a 94% likelihood of mortality [13-15].

Both indices have been widely used internationally, and several authors have proposed alternative cutoff points to optimize their predictive capacity. For instance, Eğin et al.’s study used cutoffs of 6 for the FGSI and 8 for the UFGSI, resulting in higher sensitivity and specificity than previously reported. However, limited research on the performance of these scales in Mexican populations underscores the need to identify which scale is more strongly associated with mortality in this clinical context. This would enhance the accuracy of risk stratification and support more precise decision-making for patients with FG [8-16].

This study aimed to assess the association of FGSI and UFGSI with in-hospital mortality and to explore their comparative prognostic performance in a Mexican population with FG.

## Materials and methods

This was a retrospective cohort analytical study conducted at the Hospital Regional “General Ignacio Zaragoza” (HRGIZ) ISSSTE in Mexico City. The study protocol was reviewed and approved by the Research Ethics Committee, which assigned it the Institutional Review Board (IRB) number 674-2024. Patients with a diagnosis of FG who were treated between July 2022 and February 2025 were included.

The sample was non-probabilistic and convenience-based. Inclusion criteria comprised patients over 18 years of age of both sexes with a clinically confirmed diagnosis of FG, including both survivors and deceased individuals. Clinical records had to contain complete data on the clinical, demographic, and laboratory variables necessary to calculate the predictive scales.

Exclusion criteria involved patients who died before receiving initial medical or surgical treatment and those with incomplete clinical records or significant data loss. Cases with incomplete data required for the calculation of severity indices were excluded to ensure analytical consistency.

Variables and scoring systems

Fournier Gangrene Severity Index

FGSI was calculated using nine physiologic variables at admission, namely, temperature, heart rate, respiratory rate, serum sodium, serum potassium, serum creatinine, hematocrit, leukocyte count, and serum bicarbonate. Each variable was scored from 0 to 4 according to deviation from normal values, and total FGSI was the sum of all component scores. A traditional cutoff >9 was used for mortality risk stratification, as originally described (Laor et al., 1995) [17].

Uludag Fournier Gangrene Severity Index

UFGSI includes all FGSI variables and adds two components, i.e., extent of gangrene and age (Yilmazlar et al., 2010) [13]. The extent of gangrene was classified by anatomic spread: Grade I (perineal/urogenital region), Grade II (extension to the pubis or the thigh-pelvic region), and Grade III (extension beyond the pelvic region), each weighted as 1, 2, and 6 points, respectively. Age ≥60 years added 1 point. A traditional cutoff >9 was used as described in the original UFGSI study [13].

Age-Adjusted Charlson Comorbidity Index

Comorbidities documented at admission were recorded, and the ACCI was calculated using the original Charlson methodology [18]. For analysis, ACCI was dichotomized at a cutoff ≥4, consistent with prior work [9,18]. Laboratory values were used to calculate scores corresponding to measurements obtained at hospital admission.

Statistical analysis

Quantitative variables are presented as means ± standard deviations (SDs); categorical variables are presented as n (%). Survivors and non-survivors were compared using Student’s t-test (continuous variables) and chi-square tests (categorical variables). Associations between mortality and predictors were evaluated using multivariate logistic regression models that incorporated FGSI, UFGSI, ACCI, age, and key comorbidities (diabetes, systemic arterial hypertension, chronic kidney disease, hypothyroidism, and benign prostatic hyperplasia). Models were built using both traditional cutoffs (FGSI > 9, UFGSI > 9) and adjusted thresholds (FGSI ≥ 6, UFGSI ≥ 8). For the ACCI, a cutoff point of ≥4 was used [16]. Variables included in multivariate models were selected based on clinical relevance and prior literature. Multicollinearity among predictors was assessed before model construction, and logistic regression assumptions were evaluated. Cases with missing key variables required to calculate severity indices were excluded from analysis; no imputation methods were applied. Results are expressed as odds ratios (ORs) with 95% confidence intervals (CIs). A risk was considered when OR > 1 and the 95% CI did not include the null value of 1. Analyses were conducted using SPSS version 26 (IBM Corp., Armonk, NY, USA). A two-tailed p-value <0.05 was considered statistically significant.

## Results

A total of 65 patients with a confirmed diagnosis of FG were included after excluding 16 cases due to incomplete clinical information. Most patients were male (44/65, 67.7%). The overall mean age was 59.4 years. When stratified by survival status, 18/65 patients (27.7%) died and were significantly older than survivors (64.8 ± 10.5 vs. 57.5 ± 11.4 years; p < 0.05). The most frequently identified etiology was proctologic origin (37/65, 56.9%). Regarding comorbidities, diabetes was the most prevalent (46/65, 70.8%), followed by systemic arterial hypertension (25/65, 38.5%) (Tables 1, 2).

**Table 1:** Key baseline characteristics by survival status in patients with Fournier’s gangrene (n = 65). Values are presented as mean ± SD unless otherwise specified. *: Student’s t-test.

Variable	Total (n = 65)	Alive (n = 47)	Dead (n = 18)	P-value
Age (years), mean ± SD	59.4 ± 11	57.5 ± 11.4	64.8 ± 10.5*	<0.05

**Table 2:** Sex distribution, comorbidities, and etiology (origin) of Fournier’s gangrene (n = 65). Values are presented as n (%). Percentages were calculated using n = 65 as the denominator.

Variable	Value, n (%)
Sex	
Male	44 (67.7)
Female	21 (32.3)
Comorbidities	
Diabetes	46 (70.8)
Systemic arterial hypertension	25 (38.5)
Chronic kidney disease	4 (6.2)
Cancer	2 (3.1)
Stroke	1 (1.5)
Hypothyroidism	3 (4.6)
Benign prostatic hyperplasia	5 (7.7)
Cardiovascular disease	2 (3.1)
Etiology (origin)	
Proctologic	37 (56.9)
Genitourinary	25 (38.5)
Contiguous spread	3 (4.6)

When comparing severity indices by survival outcome, mean FGSI scores were higher in non-survivors than in survivors (9.1 ± 4.0 vs. 5.8 ± 3.6; p = 0.002). Similarly, mean UFGSI scores were significantly higher among non-survivors than survivors (12.6 ± 5.1 vs. 8.4 ± 4.3; p = 0.001), indicating that both indices discriminated between mortality risk groups, with UFGSI showing greater separation (Table 3).

**Table 3:** Comparison of mean FGSI and UFGSI scores according to survival status in patients with Fournier’s gangrene. *: Student’s t-test. Quantitative data are presented as mean ± SD. FGSI = Fournier Gangrene Severity Index; UFGSI = Uludag Fournier Gangrene Severity Index

Variable	Survivors (n = 47)	Non-survivors (n = 18)	P-value
FGSI	5.8 ± 3.6	9.1 ± 4.0*	0.002
UFGSI	8.4 ± 4.3	12.6 ± 5.1*	0.001

**Figure 1:**
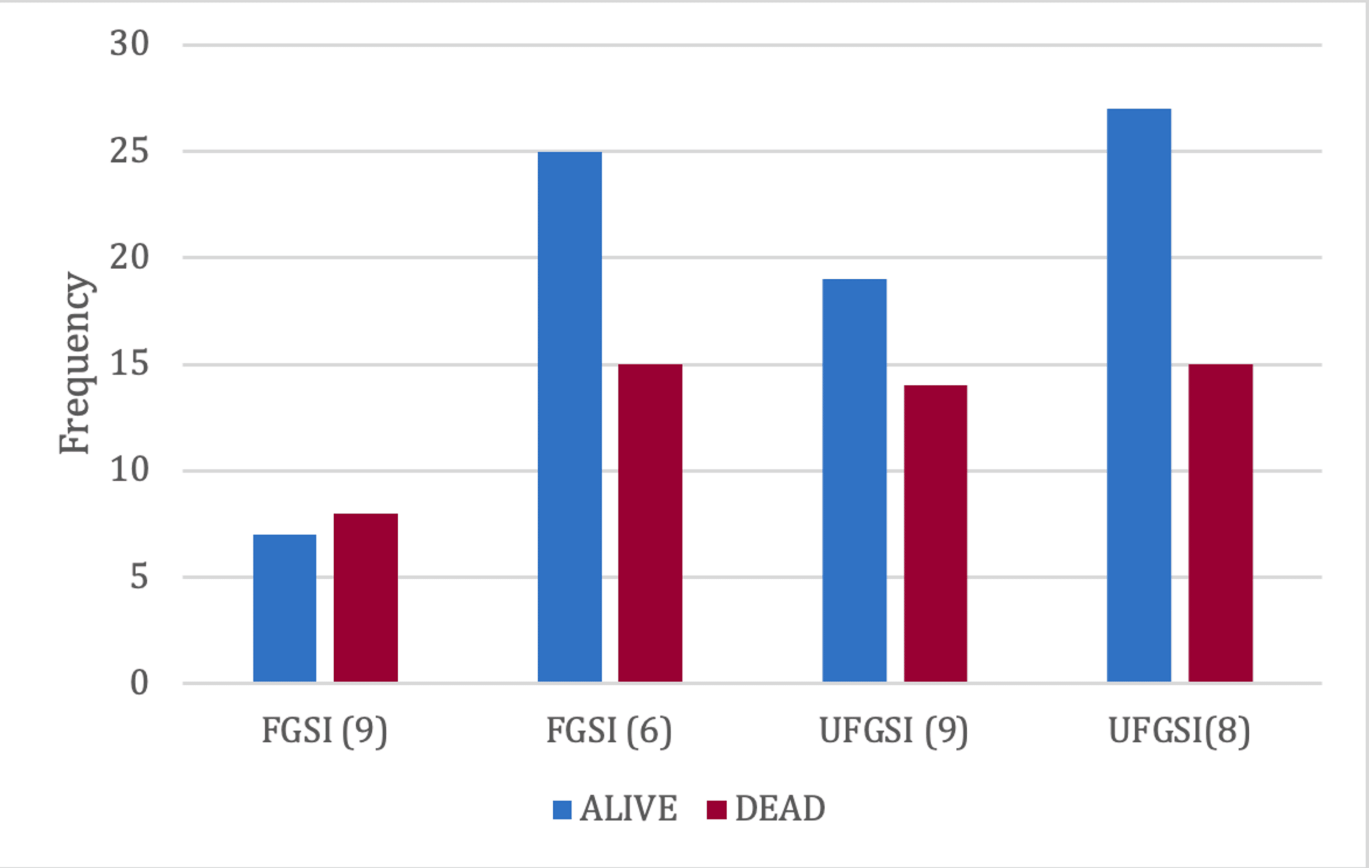
Comparison of the FGSI and UFGSI for severity assessment with cutoff adjustment. Comparison of the mortality predictive capacity of the FGSI and UFGSI through cutoff point adjustment. The numerical values in parentheses represent the clinical thresholds applied to each scale: FGSI (9) and FGSI (6) correspond to the FGSI with cutoff points of 9 and 6, respectively; while UFGSI (9) and UFGSI (8) correspond to the UFGSI with cutoff points of 9 and 8, respectively. FGSI = Fournier Gangrene Severity Index; UFGSI = Uludag Fournier Gangrene Severity Index

Using both the traditional cutoffs and those proposed by Eğin et al. [16], we observed that applying thresholds of FGSI ≥ 6 and UFGSI ≥ 8 identified the same number of deaths (Figure 1).

**Figure 2:**
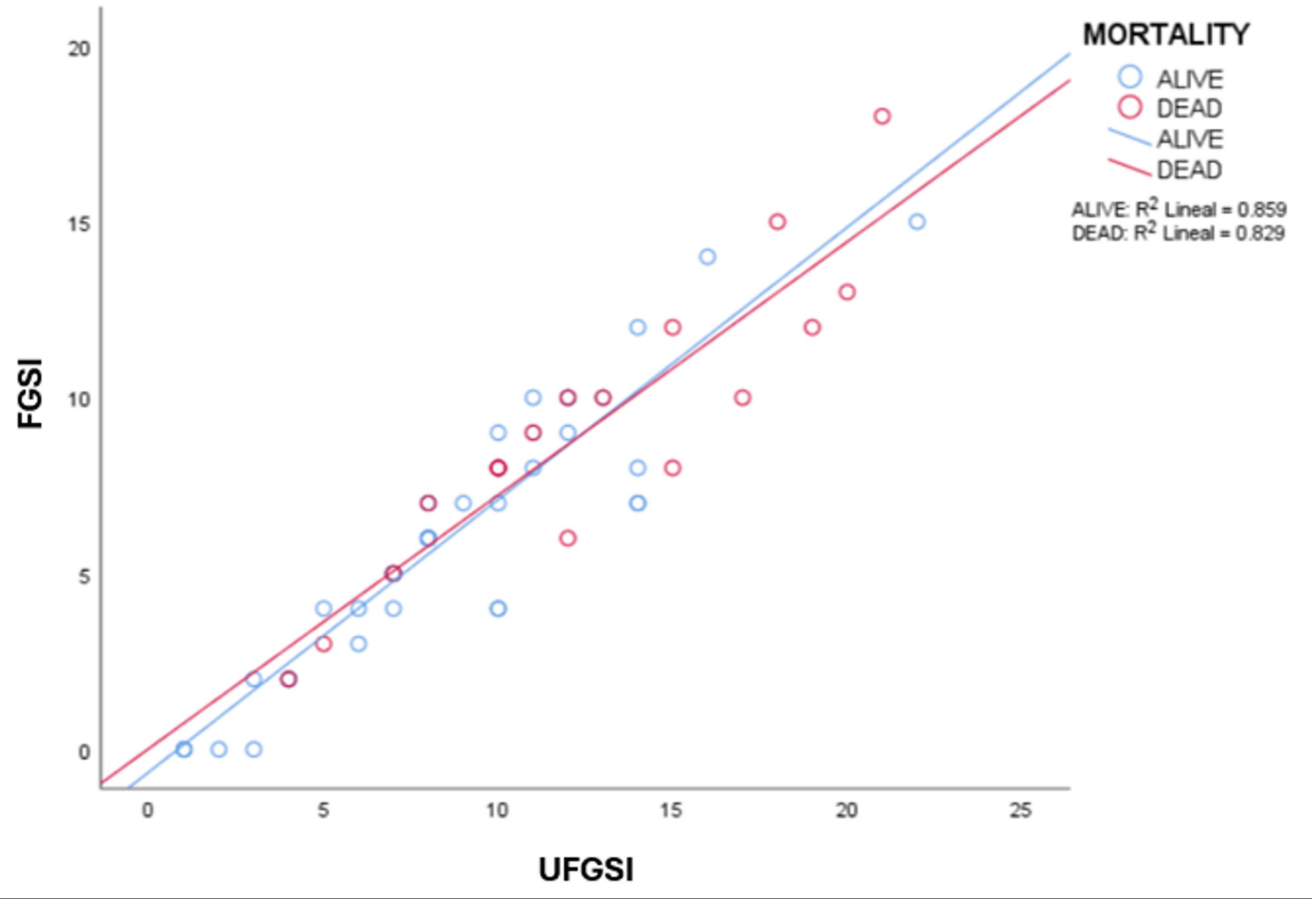
Stratified linear relationship between the UFGSI and FGSI in patients diagnosed with Fournier’s gangrene. The red line represents non-survivors (r = 0.910; r² = 0.829; p < 0.001), and the blue line represents survivors (r = 0.926; r² = 0.859; p < 0.001). FGSI = Fournier Gangrene Severity Index; UFGSI = Uludag Fournier Gangrene Severity Index

A statistically significant positive linear correlation was identified between the two indices among decedents (r = 0.91; p < 0.001). The coefficient of determination (r² = 0.829) indicated that UFGSI explained 82.9% of the variability in FGSI (Figure 2).

In multivariate logistic regression models using traditional cutoff values, both indices were independently associated with in-hospital mortality. The FGSI showed an OR of 3.70 (95% CI = 1.01-13.55; p = 0.048) and the UFGSI showed an OR of 4.70 (95% CI = 1.26-17.44; p = 0.021). Neither the ACCI nor age showed a statistically significant association in the models evaluated. Likewise, none of the individual comorbidities analyzed independently reached statistical significance, whereas both indices consistently remained the variables most strongly associated with mortality. Notably, the risk estimate for FGSI increased in the model adjusted for comorbidities (Table 4).

**Table 4:** Multivariate logistic regression for predicting mortality using FGSI, UFGSI, ACCI, and individual comorbidities. FGSI = Fournier Gangrene Severity Index (cutoff >9); UFGSI = Uludag Fournier Gangrene Severity Index (cutoff >9); ACCI = Age-Adjusted Charlson Comorbidity Index (≥4); SAH = systemic arterial hypertension; CKD = chronic kidney disease; BPH = benign prostatic hyperplasia; OR = odds ratio; CI = confidence interval

Model	Variable	OR (95% CI)	P-value
1	UFGSI	4.70 (1.26-17.44)	0.021
	ACCI	1.12 (0.21-6.15)	0.90
	Age	1.06 (0.99-1.13)	0.12
2	FGSI	3.70 (1.01-13.55)	0.048
	ACCI	1.82 (0.33-10.06)	0.50
	Age	1.04 (0.97-1.11)	0.27
3	UFGSI	4.36 (1.12-16.86)	0.033
	Diabetes	1.02 (0.26-3.95)	0.98
	SAH	1.56 (0.44-5.60)	0.49
	CKD	1.47 (0.16-13.22)	0.73
	Hypothyroidism	1.57 (0.10-24.52)	0.75
	BPH	0.53 (0.04-6.47)	0.62
4	FGSI	4.75 (1.22-18.60)	0.025
	Diabetes	1.05 (0.27-4.10)	0.95
	SAH	1.60 (0.45-5.70)	0.47
	CKD	3.26 (0.35-30.66)	0.31
	Hypothyroidism	1.83 (0.14-24.40)	0.65
	BPH	0.31 (0.02-5.56)	0.43

When adjusted cutoff points were applied (FGSI ≥ 6 and UFGSI ≥ 8), the FGSI yielded an OR of 6.01 (95% CI = 1.12-32.39; p = 0.037) only in the model adjusted for individual comorbidities, the only adjusted-threshold model to reach statistical significance (Table 5).

**Table 5:** Multivariate logistic regression for predicting mortality using adjusted scores of the FGSI and UFGSI, as well as ACCI and comorbidities. The FGSI, with a cutoff point of 6, and the UFGSI, with a cutoff point of 8. FGSI = Fournier Gangrene Severity Index (cutoff >9); UFGSI = Uludag Fournier Gangrene Severity Index (cutoff >9); ACCI = Age-Adjusted Charlson Comorbidity Index (≥4); SAH = systemic arterial hypertension; CKD = chronic kidney disease; BPH = benign prostatic hyperplasia; OR = odds ratio; CI = confidence interval

Model	Variable	OR (95% CI)	P-value
1	FGSI	3.41 (0.83-13.99)	0.08
	ACCI	1.87 (0.45-7.84)	0.39
	Age	2.40 (0.65-8.77)	0.18
2	UFGSI	3.02 (0.74-12.41)	0.13
	ACCI	1.90 (0.46-7.96)	0.38
	Age	2.57 (0.70-9.34)	0.15
3	FGSI	6.01 (1.12-32.39)	0.037
	Diabetes	1.38 (0.35-5.43)	0.65
	SAH	1.71 (0.50-5.60)	0.40
	CKD	1.60 (0.18-14.14)	0.68
	Hypothyroidism	3.74 (0.21-65.43)	0.37
	BPH	0.45 (0.04-5.39)	0.53
4	UFGSI	3.66 (0.83-16.17)	0.09
	Diabetes	1.21 (0.32-4.61)	0.79
	SAH	1.86 (0.54-6.35)	0.32
	CKD	1.74 (0.20-15.49)	0.62
	Hypothyroidism	1.59 (0.10-24.76)	0.74
	BPH	0.50 (0.04-5.91)	0.58

## Discussion

In this study, among the 65 patients diagnosed with FG, there was a clear predominance of male sex (67.7%), consistent with previous reports. Although female sex has been associated with a lower incidence of FG, possibly due to hormonal and anatomical factors facilitating better drainage of pelvic secretions, this apparent advantage contradicts findings in various studies showing higher mortality rates in women, attributed to an increased risk of developing peritonitis and retroperitonitis. However, in the present study, this association was not confirmed [6,19,20].

Regarding etiology, the proctologic origin was the most common, followed by genitourinary, and, less frequently, contiguous sources. This distribution aligns with the existing literature, which recognizes infections in the anorectal and urogenital regions, such as perianal, ischio-rectal, and scrotal abscesses, as well as trauma or invasive urological procedures, as common triggering factors [4,15].

The overall mortality rate in this study was 27.7%, which falls within the range reported in the current literature. Several studies, including meta-analyses by Tufano et al. (2023) and Seretis et al. (2025), have documented mortality rates ranging from 22.6% to 28%. Nevertheless, some series have reported substantially lower rates, up to 7.5%, highlighting the variability in prognosis depending on population characteristics and resource availability [8,21,22].

Although patients who died had a significantly higher mean age compared to survivors (64.8 vs. 57.5 years, p < 0.05), multivariate logistic regression analysis found that age was not an independent predictor of mortality. This finding contrasts with other retrospective studies, which identified age as a significant prognostic factor. This discrepancy may be attributed to the sample size, study duration, and the higher mean age of the cohort [10,16].

The main objective of this study was to evaluate the association of the FGSI and UFGSI indices with mortality. Both proved to be valuable tools, as patients with scores above 9 had nearly a fourfold increased risk of death compared to those with lower scores. Notably, UFGSI showed a stronger association with mortality, identifying 77.8% of the deceased patients. This aligns with the meta-analysis by Tufano et al. (2023), which also found that the UFGSI has higher sensitivity. However, that meta-analysis cautioned that both the FGSI and UFGSI may be impractical in routine clinical settings, prompting the search for simpler, more applicable scales. Similarly, Eğin et al. (2022) reported a sensitivity of 100% and a specificity of 68% for the UFGSI, with higher sensitivity but slightly lower specificity than Yilmazlar et al. (2010), who used the adjusted cutoff points [8,13,16].

In multivariate regression, most models incorporating adjusted cutoff points were not statistically significant, with significance observed only for the FGSI after adjustment for comorbidities. This suggests some potential utility of the proposed cutoff, but caution is warranted, as the other models did not confirm this association. Hence, these results do not definitively demonstrate that the adjusted cutoff points outperform the traditional ones, highlighting the need for larger studies to validate their prognostic utility.

Regarding comorbidities, diabetes was the most prevalent. This condition has been associated with increased susceptibility to severe infections due to immune dysfunction caused by chronic hyperglycemia. Several studies have linked uncontrolled diabetes to higher mortality in FG. However, in our study, despite its high prevalence, diabetes was not associated with mortality in multivariate models. This aligns with findings by Ioannidis et al. (2017) and may partly be explained by the lack of differentiation between controlled and uncontrolled diabetes, an area for future research [4,15,23-25].

The ACCI was used to assess the overall impact of comorbidities on mortality. However, in our study, this index was not associated with mortality among patients with FG, unlike previous studies reporting a significant association between ACCI and adverse outcomes. This discrepancy could be attributed to differences in sample size, the cutoff scores used to classify it as a risk factor, the clinical characteristics of the studied populations, or the type of analysis performed [9,25,26].

An important confounder not captured in this study is the time from diagnosis to initial surgical debridement. Early aggressive surgical intervention is widely recognized as a cornerstone of management in FG, and delays have been consistently associated with increased mortality. Due to the retrospective nature of our dataset and incomplete documentation, this variable could not be reliably analyzed. Future prospective studies should incorporate it as a key variable to better characterize its impact on prognostic performance.

It is essential to acknowledge the limitations of this study. The retrospective design may have resulted in incomplete capture of relevant clinical data and may introduce selection bias. We did not perform a formal comparison between included and excluded patients because data were incomplete in excluded cases, which may further limit generalizability. Although the sample size was acceptable for an exploratory analysis, it may have limited the statistical power to detect more subtle associations. The wide CIs observed across several regression models reflect limited precision, likely related to the modest sample size and number of events, and results should therefore be interpreted with caution. Given the relatively small number of mortality events, multivariate models including multiple predictors may be susceptible to overfitting, potentially inflating effect estimates and reducing model stability. Formal discrimination analyses, such as receiver operating characteristic curves or area under the curve comparisons, were not performed, limiting the ability to draw definitive conclusions about comparative predictive performance. Accordingly, findings should be interpreted as exploratory and hypothesis-generating, and more parsimonious modeling or penalized regression approaches should be considered in future studies. Finally, the lack of differentiation between controlled and uncontrolled chronic conditions, such as diabetes, may have influenced the assessment of their impact on outcomes.

## Conclusions

The FGSI and UFGSI were associated with in-hospital mortality in patients with FG. In this cohort, UFGSI showed a stronger association with adverse outcomes when traditional cutoff values were applied, suggesting potential clinical value for early risk stratification. From a clinical perspective, the use of these indices may support risk assessment and inform decision-making during in-hospital management. Although adjusted cutoff points did not demonstrate a consistent advantage, their evaluation provides relevant insights and highlights the need for larger prospective studies to further validate and refine their prognostic utility. These findings should be interpreted within the context of the study’s retrospective design and limited sample size.
